# Plant Defence Metabolite Detoxification by a Pathogenic Fungus Opens Necrotic Tissue to Diverse Bacteria in *Arabidopsis thaliana*

**DOI:** 10.1111/1758-2229.70401

**Published:** 2026-08-19

**Authors:** Shubhangi Sharma, Jingyuan Chen, Matthew Agler

**Affiliations:** ^1^ Plant Microbiosis Lab, Institute for Microbiology Friedrich Schiller University Jena Jena Germany; ^2^ Faculty of Arts and Sciences, Beijing Normal University Zhuhai China; ^3^ Department of Biochemistry Max Planck Institute for Chemical Ecology Jena Germany; ^4^ Cluster of Excellence Balance of the Microverse Friedrich Schiller University Jena Jena Germany

## Abstract

Foliar necrotic fungal pathogens pose a constant threat to plant health. Their virulence can be affected by both the host immune system, as well as diverse host‐associated bacteria. Generally, how these factors interact is not well studied, but may provide insights toward sustainable plant protection. *Sclerotinia sclerotiorum* (Ssc) is a devastating, broad host range necrotrophic pathogen with limited control options. Here, we find that necrotic Ssc lesions strongly enrich diverse bacterial taxa. *Arabidopsis thaliana* , like other Brassica plants including crops, produce and store large amounts of aliphatic glucosinolates (aGLS). Upon Ssc invasion and tissue damage, myrosinase enzymes transform aGLS into toxic metabolites, including aliphatic isothiocyanates (aITCs). Adapted Ssc strains can detoxify aITCs by hydrolyzing them with the enzyme SaxA, which is important for its virulence. Here, we find that this detoxification is critical for bacterial enrichment in necrotic lesions, since a SaxA‐deficient strain does not alter the bacteriome. Additionally, our results suggest that both aliphatic and other GLSs likely contribute specific effects to this phenomenon. Thus, we demonstrate a mechanism in which the interaction of host and pathogen factors together shape a pathogen‐associated bacteriome, providing directions to better understand interactions of host bacteria with foliar necrotic pathogens.

## Graphical abstract

*Sclerotinia sclerotiorum*, a devastating necrotrophic fungal pathogen of many crops, recruits a specific microbiome to necrotic, diseased leaf tissue. The interaction between plant formation and fungal degradation of aliphatic glucosinolate‐derived defence metabolites nearly completely controls this recruitment.

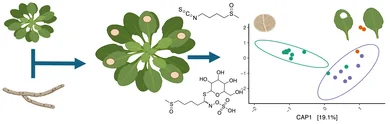

## 1 Introduction

Most plant tissues are colonized by a variety of microorganisms, including bacteria, fungi, and other eukaryotes that play critical roles in plant health and productivity. Bacterial diversity is of particular interest because of their essential roles in protecting plants, for example from detrimental environmental pathogens (Duran et al. 2018; Paasch et al. 2023). Mechanisms of protection are both direct and indirect, including antimicrobial secretion, resource competition, and the activation of the plant's own natural defences (Vannier et al. 2019).

Although they are key to utilizing protective bacterial diversity, so far the mechanisms of tripartite plant‐pathogen‐bacteriome interaction dynamics are not well understood.

In leaves, bacterial diversity is largely controlled by filtration effects exerted by plant host factors and the environment. (Wagner et al. 2016). Importantly, these factors can act strongly via inter‐kingdom microbe‐microbe interactions (Agler et al. 2016). For example, foliar fungal biotrophic pathogens cause strong changes to leaf bacterial community diversity upon infection by increasing the abundance of diverse bacterial taxa (Duran et al. 2021; Jakuschkin et al. 2016). Bacteria recruited to leaves during pathogen infection can influence disease outcome (Goossens et al. 2023), so it is important to understand these processes. It has been suggested that rearrangements of host defence and metabolism by pathogens could play an important role, but the mechanisms have in most cases not yet been uncovered.

Plant specialized metabolites play important roles in plant protection especially against herbivores and nonhost pathogens. A well‐studied defence system in the Brassicaceae, including important crops and the model plant *Arabidopsis thaliana* is the aliphatic glucosinolate (aGLS)—myrosinase system. aGLSs are amino acid‐derived secondary defence metabolites which are not by themselves biologically active and are stored intact at high levels within the vacuoles of specialized leaf cells. Upon tissue damage, they come into contact with myrosinase enzymes (β‐thioglucoside glucohydrolase) and are rapidly broken down into diverse toxic metabolites, commonly known as the “mustard oil bomb” (Halkier and Gershenzon 2006). In the model *A. thaliana* genotype Col‐0, the main breakdown products are aliphatic isothiocyanates (aITCs). These highly reactive compounds are well‐recognized for their antimicrobial activity against both bacterial and fungal plant pathogens (Chen et al. 2020; Fan et al. 2011).

Host‐adapted bacterial and fungal pathogens of *A. thaliana* express ITC hydrolases (metallo‐β‐lactamase enzymes) known as SaxA (“survival in Arabidopsis extracts”) enzymes. These help them handle the massive release of ITCs upon tissue destruction by hydrolyzing them to non‐toxic amines. Pathogens without these hydrolases are compromised in virulence in *A. thaliana* (Chen et al. 2020; Fan et al. 2011). Besides pathogens, ITCs like sulforaphane in the *A. thaliana* genotype Col‐0 are also toxic to bacteria that colonize healthy plant leaves. Indeed, a subset of these bacteria express similar activity that can detoxify ITCs (Unger et al. 2024). Thus, both bacterial and fungal pathogens modify the *A. thaliana* leaf chemical environment upon invasion by instigating glucosinolate hydrolysis into toxic products, as well as by detoxifying those products.

*Sclerotinia sclerotiorum* de Bary (Ssc) is a highly destructive fungal necrotrophic pathogen with a wide host range, including important Brassicaceae crop plants. It expresses a SaxA protein that increases its virulence on *A. thaliana* by detoxifying GLS breakdown products. Little is known about how Ssc influences the leaf microbiome or the role of ITCs in this process. We hypothesized that during leaf necrosis, ITC release and its subsequent hydrolysis would modulate assembly of a pathogen‐associated bacterial community. To test this hypothesis, we studied the effect of Ssc infection on local and systemic leaf bacteriomes of *A. thaliana* Col‐0. To address potential correlative limitations, the experiment integrated two genetic modifications, one in the host and one in the pathogen, that each independently manipulates the GLS/ITCs/SaxA interaction system. Specifically, we looked at bacteriomes in the Col‐0 WT and its aGLS‐free *myb28/myb29* mutant (Sønderby et al. 2007), each infected with either the necrotrophic fungus *S. sclerotiorum* (Ssc WT) or the Ssc Δ*SaxA* mutant (Chen et al. 2020), which cannot hydrolyze ITCs. This dual‐genetic framework allowed for a direct evaluation of how ITC hydrolysis can modulate bacteriome assembly in *A. thaliana* Col‐0.

## 2 Materials and Methods

### 2.1 Fungal Strains and Growth Conditions

The *Sclerotinia sclerotiorum* strain 1980 wild‐type (Ssc WT) and the knockout mutant strain Ssc Δ*SaxA* (Chen et al. 2020) were routinely cultured on potato dextrose agar (PDA) at 24°C for 4 days. In order to prepare fungal inoculum, the fungus was cultured in liquid potato dextrose broth (PDB) at 24°C and gentle shaking at 120 rpm for 3 days. The fungal biomass was strained in a sterile nylon mesh and washed with autoclaved miliQ water until the flow‐through was clear to remove all PDB. The washed fungal biomass was crushed in a blender along with fresh 40 mL of PDB at the slowest speed for 40s. The number of fungal hyphal fragments were counted in a Thoma cell counting chamber and the fungal inoculum was adjusted to 2 × 10^5^ fragments per mL using PDB.

### 2.2 Plant Cultivation, Inoculation and Sampling

*Arabidopsis thaliana* ecotype Columbia (Col‐0) and the *myb28/29* double knockout mutant (in the Col‐0 background) which does not produce aliphatic GLS were used. Seeds were vernalised in 0.1% agarose at 4°C for 5 days. A standard laboratory potting soil was made by mixing 4 L of Floraton 3 potting soil (Floragard), 2 L perlite (0‐6 mm Perlite Perligran, Knob), and 25 g Substral Osmocote garden flower fertilizer (Celaflor). This soil was supplemented with a soil slurry inoculum made by dissolving 15 g of dried garden soil (collected from a common garden outside our laboratory) in 100 mL of miliQ water. Two parts soil slurry were used to wet four parts of dry laboratory potting soil. The plants were grown on an LED light shelf under 14‐h days at about 260 μmol·m^−2^·s^−1^ for four weeks.

### 2.3 Ssc Inoculation and Sampling

**FIGURE 1:**
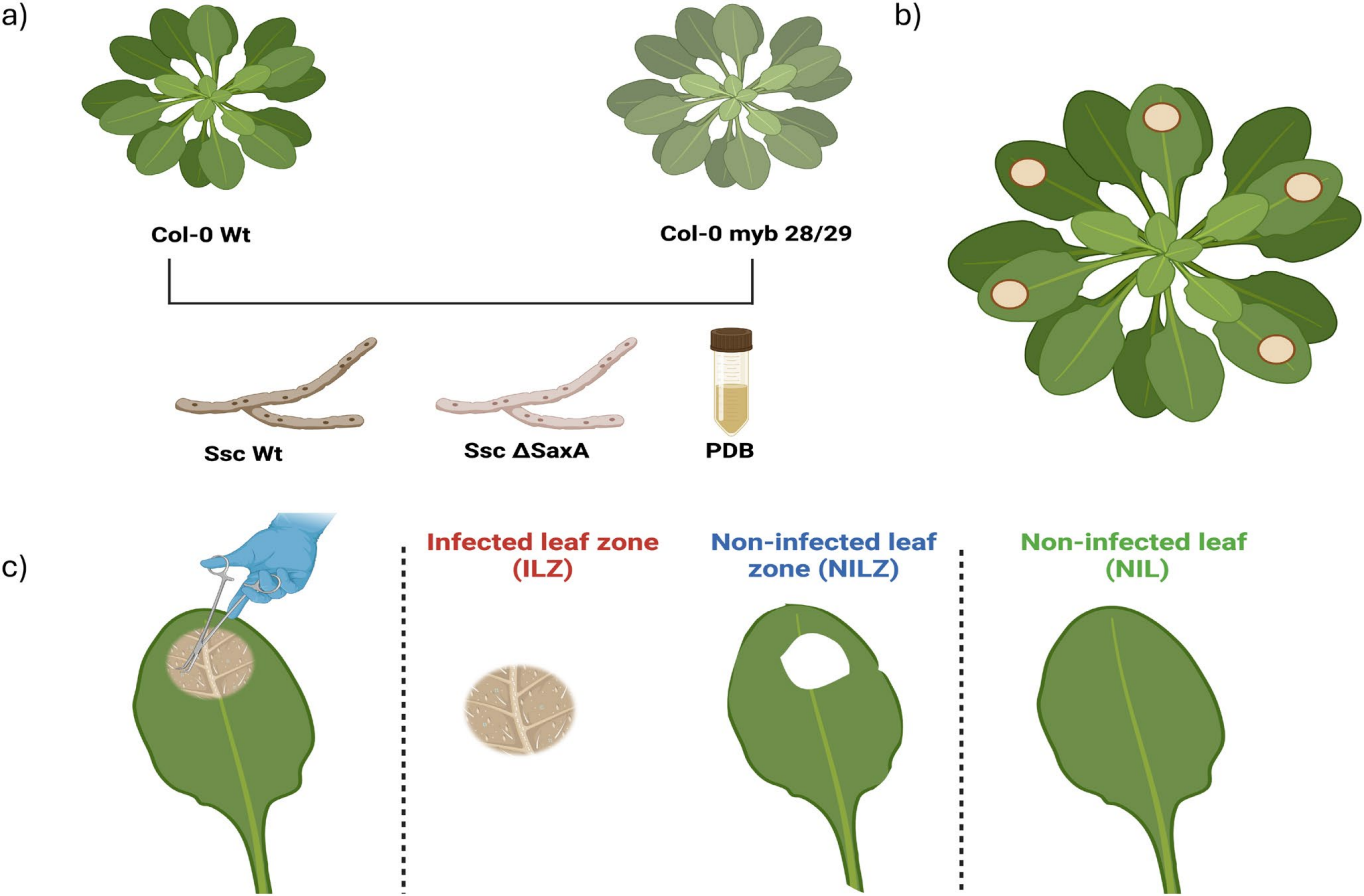
Inoculation and sampling strategy for *S. sclerotiorum* infected *A. thaliana* leaves. (A) *A. thaliana* Col‐0 and myb28/29 mutant plants grown in a natural soil‐supplemented substrate were drop‐inoculated with Ssc WT, Ssc Δ*SaxA* or a PDB control. (B) Leaves inoculated with Ssc developed necrotic lesions at the site of inoculation. (C) Inoculated leaves were dissected so that bacterial communities could be characterized in necrotic tissue (ILZ), non‐necrotic tissue of the same leaf (NILZ) and in systemic tissue (NIL). PDB‐inoculated control plants were dissected similarly. A more detailed version of the figure is provided in Figure S1.

Four‐week old *A. thaliana* Col‐0 or *myb28/29* plants were infected with *S. sclerotinia* (WT or Δ*SsSaxA*) hyphal suspension or inoculated with a PDB only control (Figure 1A). Ten individual plants of each genotype were randomly assigned to each treatment. Five leaves per plant were inoculated with a 5 μL drop. The plants were covered with a lid to increase humidity for successful infection. After development of symptoms (36–38 h, (Figure 1B)), three tissues were taken from each plant: the necrotic zone of the leaf constituting the infected leaf zone (ILZ), the remaining leaf tissue constituting the non‐infected leaf zone (NILZ), and non‐inoculated leaves (NIL) as systemic tissue. Each sample consists of combined tissues collected from three leaves from a single plant. The PDB control plants were treated the same, but the “ILZ” corresponds to the spot where PDB was inoculated and there was no necrotic tissue (Figure 1C). All samples were flash‐frozen using liquid nitrogen and stored at −80°C.

### 2.4 DNA Extraction and 16S rRNA Gene Amplicon Library Preparation and Sequencing

DNA was extracted from all the samples using a well‐established method. Briefly, the frozen plant tissue was lysed using a bead beater for 30 s at 1400 rpm. To the lysed plant tissue, 150 μL of 2× cetyl trimethylammonium bromide (CTAB) buffer (2% cetyltrimethylammonium bromide, 1% polyvinylpyrrolidone, 100 mM Tris–HCl, 1.4 M NaCl, 20 mM EDTA) was added followed by a ten‐minute incubation at 65°C. The supernatant from the CTAB extraction was mixed with 200 μL of ice‐cold phenol‐chloroform‐isoamylalcohol (25:24:1), centrifuged at top speed for 5 min, then the supernatant was precipitated overnight at −20°C in an equal volume of isopropanol. After removing the isopropanol, the pellet was washed twice with 80% ethanol, air dried and resuspended in Tris–HCl (10 mM, pH 8).

The extracted DNA was used as a template in a 2‐step 16S rRNA gene amplicon sequencing protocol modified to quantify bacterial loads via host‐associated microbe PCR (hamPCR) (Lundberg et al. 2021). In short, 16S amplification was performed together with amplification of a single copy host plant gene (GI = *GIGANTEA* gene, referred to as “GI gene”). ZymoBIOMICS Microbial Community DNA Standard II (ZymoResearch, Freiburg, Germany; referred to as “ZymoMix”) was used as positive control in three reactions, and eight negative controls included a no template PCR control, two reactions with CTAB DNA extraction buffer, two reactions with TRIS buffer that was used to resuspend DNA, and three reactions with nuclease free water. In a first 5‐cycle PCR, samples were amplified using 341 F/799 R “universal” 16S rRNA primers and GI primers, both modified with an overhang sequence. The reaction included blocking oligos to reduce plastid 16S amplification (Mayer et al. 2021). The PCR product was enzymatically cleaned and used as template in a second, 35‐cycle PCR to add index barcodes and sequencing adapters using primers that bound to the overhang region. PCR products were cleaned up with magnetic beads and libraries were quantified using PicoGreen (1:200 diluted stock, Quant‐iT PicoGreen, ThermoFisher) in a qPCR machine (qTower3, JenaAnalytik, Jena, Germany). Samples were pooled according to their normalized fluorescence relative to the highest fluorescent sample. The pool was further processed to increase the fraction of 16S relative to GI, as recommended in the original protocol. Libraries were sequenced on an Illumina MiSeq instrument for 600 cycles. PCR reaction details and primer sequences are previously described in (Unger et al. 2024).

### 2.5 Data Analysis and Statistical Methods

The amplicon sequencing data was split into samples based on indices and adapter sequence trimmed from distal read ends using Cutadapt 3.5 (Martin 2011). We then clustered amplicon sequencing data (forward reads only as they were much higher quality) into amplicon sequencing variants (ASVs) using dada2 (Callahan et al. 2016). Chimeric sequences were removed and a sequence table retrieved from the data. Taxonomy was assigned to the final set of ASVs using the Silva 16S rRNA (v 138.1) database (Quast et al. 2013). The database was supplemented by adding the *A. thaliana* GI gene sequence.

Further filtering and quality control was performed using the R package phyloseq (McMurdie and Holmes 2013). First, the data was filtered to remove reads derived from mitochondrial or chloroplast 16S, then was split into bacterial 16S and host GI gene sets. The positive and negative controls were then checked. The positive controls looked as expected. After removing ASVs derived from the positive controls, of 11 controls (three positive controls and eight negative: one no template PCR control, two with the TRIS buffer that was used to resuspend DNA, two with CTAB DNA extraction buffer that was carried through the process and three nuclease free water controls), one positive control and one TRIS negative control contained contamination. Thus, only a small amount of contamination is expected in the samples and given the replication the results should still be very robust. Based on this analysis, we decided to keep samples with at least 100 bacterial reads (the average total read depth was ~14,000 reads/sample and most had much more than 100 bacterial reads, but as expected for leaf samples, samples with low bacterial loads contain many plant chloroplast and mitochondria reads that get filtered out).

To generate each figure, the quality controlled data was first subset to the samples of interest. All analyses were performed after grouping the ASVs at the genus level. Alpha diversity parameters were measured using the function “estimate_richness” and plotted in a boxplot. A *t*‐test was used to test for significant differences. For beta diversity analyses, the abundance data was first adjusted for the number of host GI reads to reflect absolute abundances. Next, bray‐curtis distances between samples were calculated with the function “distance” and a constrained analysis of principle coordinates was performed with function “ordinate,” constrained for the tissue type (ILZ, NILZ or NIL). A PERMANOVA analysis was performed using the function “adonis2” to evaluate significance of the tissue type on the bray‐curtis distances with 1000 permutations. Finally, the ordination was plotted using the function “plot_ordination”. An estimate of bacterial load was calculated for each sample by dividing the sum of bacterial reads by the bacterial reads + GI reads. These were square‐root transformed before plotting and a *t*‐test was used to check for significance between treatments. To test for differential abundance of the bacterial taxa between leaf tissues for a given treatment, we used DESeq2 (Love et al. 2014). Because some treatments had far more significant taxa than others, the cutoff *p*‐value was lowered for these treatments to only show a reasonable number of taxa. The *p*‐values used are described in the corresponding Supporting Information. The log‐transformed abundances of taxa identified by DESeq at the *p*‐value cutoff were plotted in boxplots bubble plots for visual inspection.

## 3 Results

### 3.1 *S. sclerotinia* Infection Alters the Leaf Bacteriome by Enriching Diverse Bacteria in Necrotic Tissue

**FIGURE 2:**
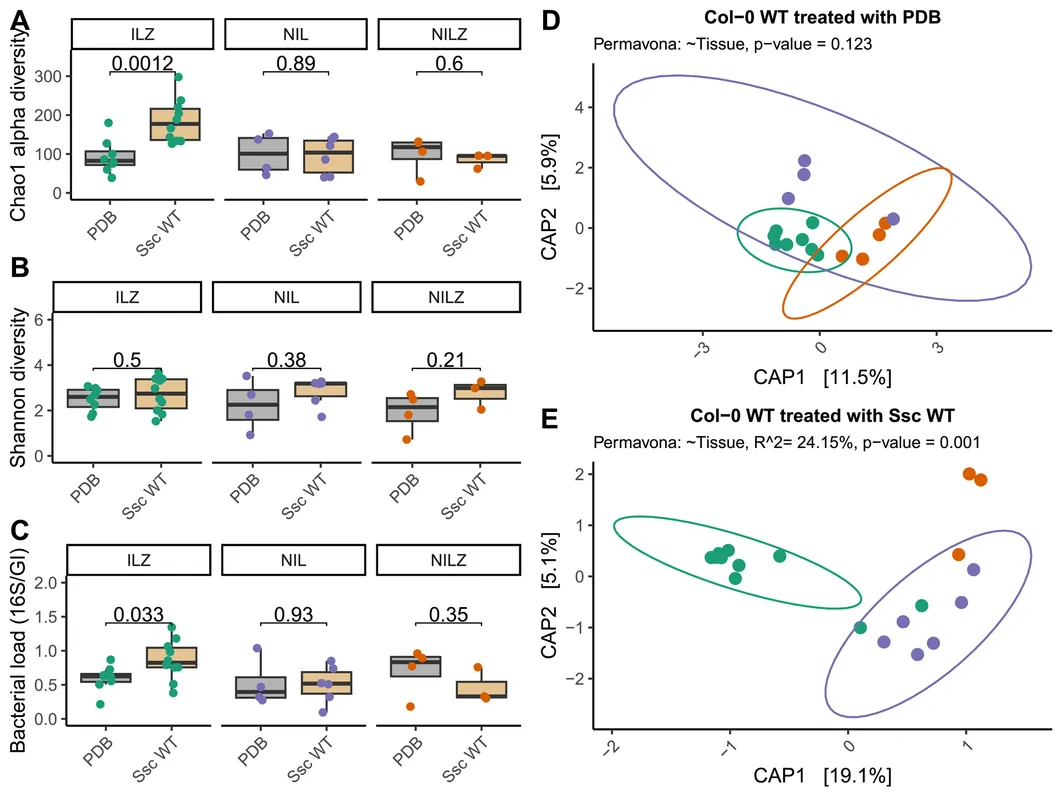
Bacterial community analysis of *A. thaliana* Col‐0 WT infected with *S. sclerotiorum* WT. Measurements of leaf bacterial community composition of different leaf tissues (ILZ, NILZ and NIL—Defined in Figure 1) infected with Ssc WT. Alpha diversity results are based on 16S rRNA gene amplicon sequencing data, grouped at the genus level, while load and beta diversity results are the same data scaled to reflect absolute abundance (hamPCR, see methods). Each point represents one leaf sample and is coloured according to the leaf tissue: Green: ILZ, Purple: NIL, Orange: NILZ. (A) Chao1 estimate of total alpha diversity, (B) Shannon Index and (C) Bacterial load in tissues from Ssc‐infected plants (tan bars) compared to the control PDB treatment (grey bars). *p*‐values are the result of a *t*‐test comparing the treatments. (D) and (E) Constrained analysis of principal coordinates showing the effect of tissue type on the bacterial community structure, based on bray‐curtis distances. PERMANOVA values reflect the result of 1000 permutations of the data.

To test whether disease caused by the necrotrophic fungal pathogen *S. sclerotinia* shapes bacterial communities inhabiting *A. thaliana* Col‐0 leaves, we infected five leaves of four‐week‐old plants with hyphal fragments of Ssc WT suspended in PDB medium or with only PDB as a control (Figure 1). Infection of Col‐0 WT by Ssc WT increased the estimated total bacterial diversity in necrotic tissue (the ILZ) compared to PDB treatment (ILZ, Chao1 *p* = 0.012) but did not affect the evenness in the community (Shannon Index, *p* = 0.5) (Figure 2A,B). Additionally, the ILZ of infected plants exhibited a significantly increased bacterial load (16S/GI *p* = 0.033) (Figure 2C). In comparison, there was no effect on diversity or bacterial loads in either the non‐infected part of the treated leaf (NILZ) nor in a systemic healthy leaf (NIL). Similarly, the Ssc WT treatment resulted in a significant shift in the bacterial community structure in the ILZ, with tissue type explaining about 24% of variation in the bacterial community. The control PDB treatment had no significant effect (Figure 2D,E, PERMAVONA *p* = 0.001 and *p* = 0.123, respectively). Overall *S. sclerotinia* infection significantly altered the local bacterial community structure, increasing total diversity, and elevated bacterial load specifically within necrotic tissue (ILZ), while having no systemic effects on distal (NIL) or non‐infected leaf zones (NILZ).

### 3.2 Fungal ITC Degradation Shapes Bacterial Enrichment in the Ssc Necrotic Zone

**FIGURE 3:**
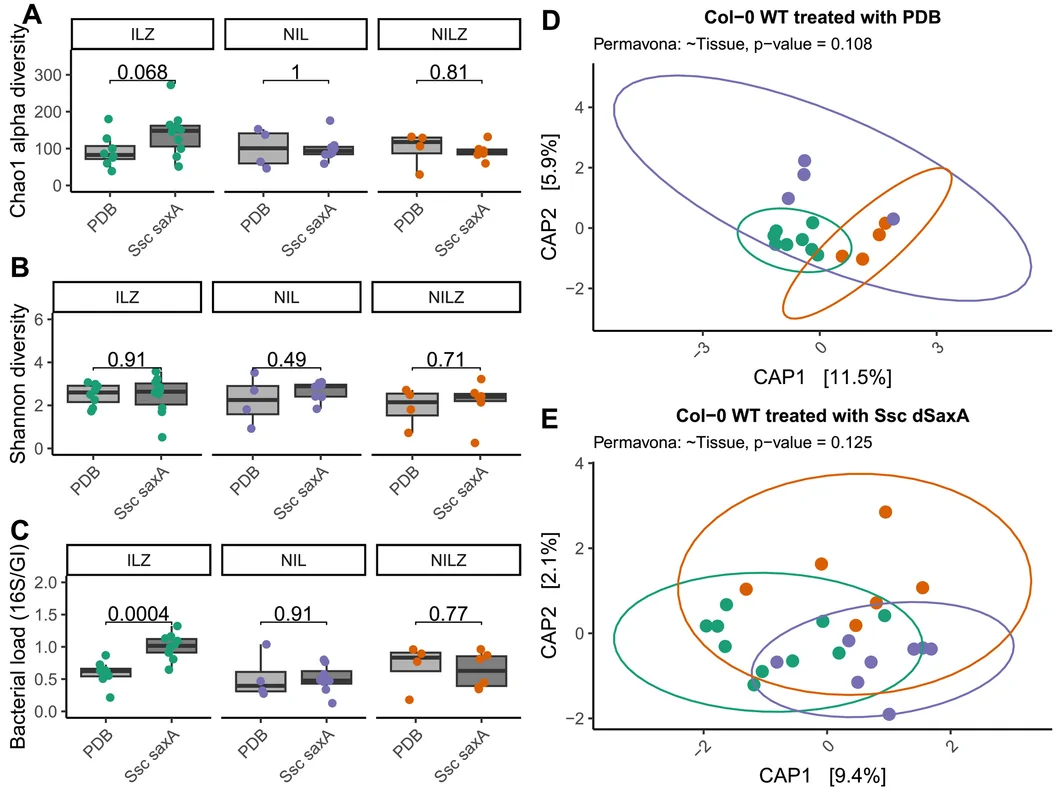
Bacterial community analysis of *A. thaliana* Col‐0 WT infected with the *S. sclerotiorum* Δ*SaxA* mutant. Measurements of leaf bacterial community composition of different leaf tissues (ILZ, NILZ and NIL—Defined in Figure 1) infected with Ssc Δ*SaxA* mutant. Alpha diversity results are based on 16S rRNA gene amplicon sequencing data, grouped at the genus level, while load and beta diversity results are the same data scaled to reflect absolute abundance (hamPCR, see methods). Each point represents one leaf sample and is coloured according to the leaf tissue: Green: ILZ, Purple: NIL, Orange: NILZ. (A) Chao1 estimate of total alpha diversity, (B) Shannon Index and (C) Bacterial load in tissues from Ssc‐infected plants (tan bars) compared to the control PDB treatment (grey bars). *p*‐values are the result of a *t*‐test comparing the treatments. (D) and (E) Constrained analysis of principle coordinates showing the effect of tissue type on the bacterial community structure, based on bray‐curtis distances. PERMANOVA values reflect the result of 1000 permutations of the data.

To assess how fungal ITC degradation (SaxA activity) contributes to altering the bacterial community in leaves infected with the fungal pathogen Ssc, we next infected *A. thaliana* Col‐0 genotype plants with the Ssc Δ*SaxA* mutant. This strain is impaired in the detoxification of ITCs. The experimental setup was identical to before. After infection with Ssc Δ*SaxA*, only a tendency for an increase in estimated total bacterial diversity was observed in the ILZ (Chao1 *p* = 0.068) with no effect on systemic tissues (NILZ or NIL) (Figure 3A,B). The bacterial load, on the other hand, was still strongly increased in the ILZ of Ssc Δ*SaxA*‐infected leaves (Figure 3C, *p* = 0.0004). Despite these changes, Ssc Δ*SaxA* infection did not result in a more significant shift in beta diversity than PDB treatment (Figure 3D,E, PERMANOVA, *p* = 0.125).

### 3.3 SaxA Affects Bacterial Diversity in the ILZ Even in the Absence of Aliphatic Glucosinolates

**FIGURE 4:**
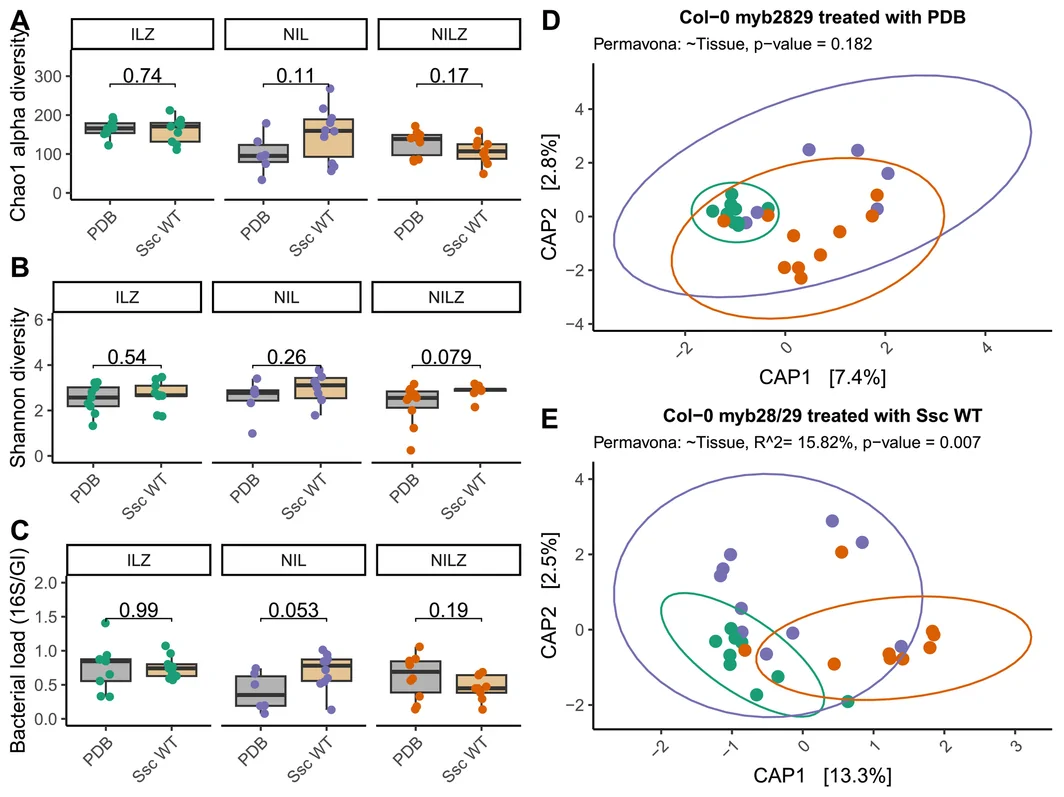
Bacterial community analysis of *A. thaliana* Col‐0 *myb28/29* genotype infected with *S. sclerotiorum* WT. Measurements of leaf bacterial community composition of different leaf tissues (ILZ, NILZ and NIL—Defined in Figure 1) infected with Ssc *WT*. Alpha diversity results are based on 16S rRNA gene amplicon sequencing data, grouped at the genus level, while load and beta diversity results are the same data scaled to reflect absolute abundance (hamPCR, see methods). Each point represents one leaf sample and is coloured according to the leaf tissue: Green: ILZ, Purple: NIL, Orange: NILZ. (A) Chao1 estimate of total alpha diversity, (B) Shannon Index and (C) Bacterial load in tissues from Ssc‐infected plants (tan bars) compared to the control PDB treatment (grey bars). *p*‐values are the result of a *t*‐test comparing the treatments. (D) and (E) Constrained analysis of principle coordinates showing the effect of tissue type on the bacterial community structure, based on bray‐curtis distances. PERMANOVA values reflect the result of 1000 permutations of the data.

To test whether the observed patterns can be explained by detoxification of aITCs alone, we performed infections on the *A. thaliana* Col‐0 *myb28/29* mutant, which is completely impaired in the biosynthesis of aGLSs. If aITCs were the main factor, we would expect to see strong bacterial enrichment in lesions of both Ssc WT and Ssc Δ*SaxA*. During infection of Col‐0 *myb28/29* with Ssc WT there was no significant effect in the ILZ or in systemic tissue on either bacterial diversity or loads (Figure 4A–C). There was, however, a significant effect of the infection on beta diversity, with tissue type explaining about 15.8% of variation in the bacterial community during Ssc WT infection (Figure 4D,E, PERMAVONA, *p* = 0.007). In contrast, lesions of Col‐0 *myb28/29* infected with Ssc Δ*SaxA* showed a small decrease in alpha diversity but no significant effect on bacterial loads (Figure S2A,C) and no significant effect on beta diversity (Figure S2D,E, *p* = 0.43). Taken together, our data are consistent with a scenario in which fungal detoxification of aITCs in necrotic lesions by SaxA plays a positive role in shaping bacterial communities, since effects of Ssc WT infection were stronger in Col‐0 WT than in Col‐0 *myb28/29*. Some taxa appear to be consistently recruited to these lesions compared to surrounding tissue. On the other hand, SaxA was still relevant in the Col‐0 *myb28/29* background, where aITCs cannot be produced, suggesting it has effects beyond aITCs.

### 3.4 SaxA‐Mediated ITC Degradation Enriches Diverse Bacterial Taxa

**FIGURE 5:**
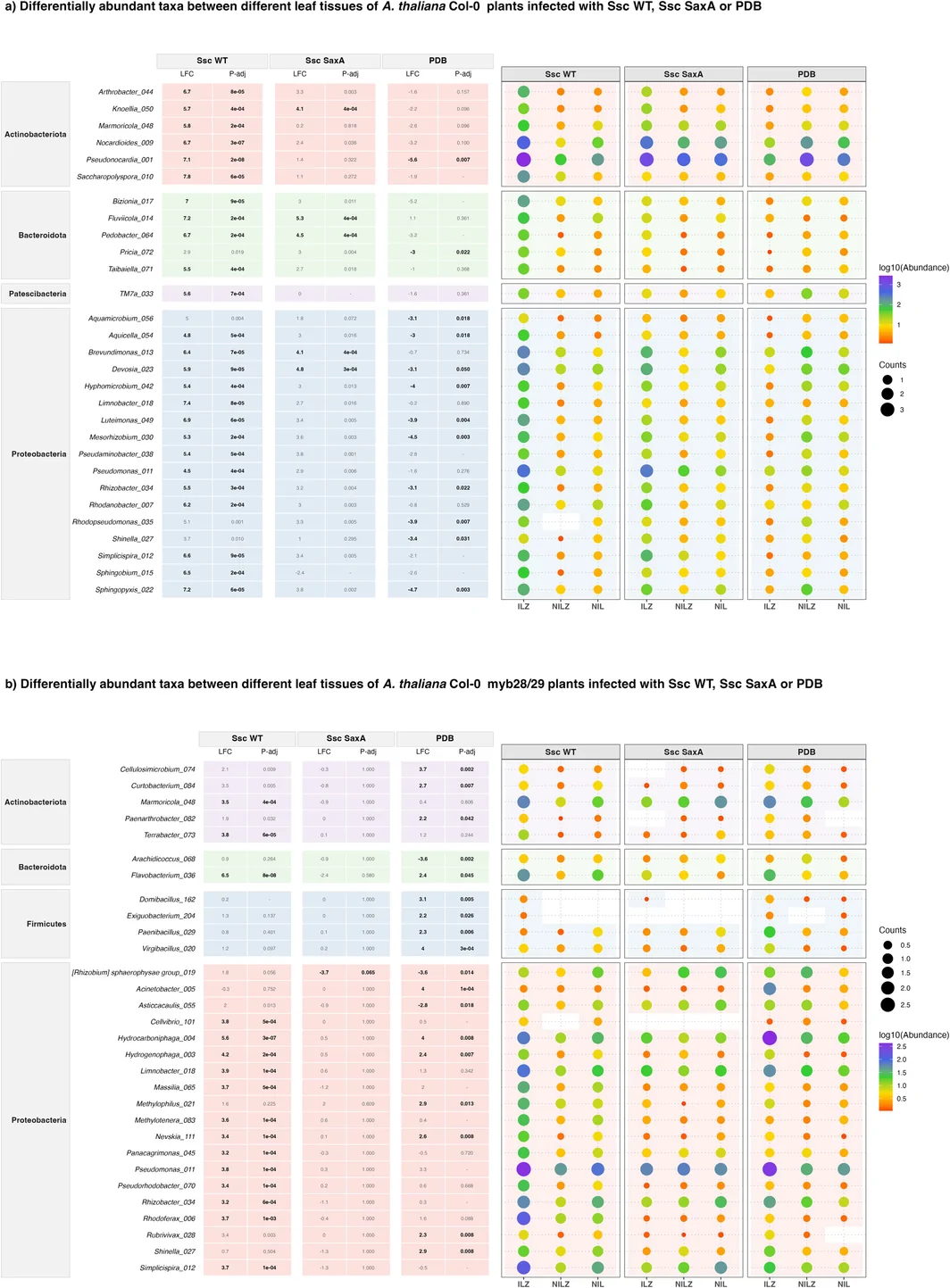
Analysis of differential abundance of bactrial taxa between the ILZ and NILZ. DESeq2 analysis based on genus‐level 16S rRNA amplicon sequencing data, scaled to reflect absolute abundance (hamPCR, see methods). (A) Differentially abundant taxa in *A. thaliana* Col‐0 plants infected with Ssc WT, *Ssc* Δ*SaxA* or PDB. NIL is also plotted for comparison. The genera predicted to be differentially abundant taxa between the ILZ and NILZ were recovered at the alpha *p*‐value threshold of 0.001 for *Ssc* WT and *Ssc* SaxA, and 0.05 for PDB. (B) Differentially abundant taxa in *A. thaliana* Col‐0 myb28/29 plants infected with Ssc WT, *Ssc* Δ*SaxA* or PDB. The genera predicted to be differentially abundant taxa between the ILZ and NILZ were recovered at the alpha *p*‐value threshold was 0.001 for *Ssc* WT, 0.1 for *Ssc* SaxA, and 0.05 for PDB. NIL is also plotted for comparison.

In *A. thaliana* Col‐0 WT leaves, *Ssc* WT infection leads to a significantly higher bacterial diversity in the necrotic leaf tissue (ILZ) compared to the non‐infected leaf tissue (NILZ). Differential abundance analysis revealed that 25 bacterial genera across the Actinobacteriota, Bacteroidota, Patescibacteria, and Proteobacteria phyla were significantly enriched in the necrotic ILZ tissue versus the adjacent NILZ tissue during *Ssc* WT infection (*p* < 0.001, Figure 5A). This localized enrichment was substantial, with all identified taxa exhibiting a log2FC of at least 4.5. In contrals, the *SaxA*‐deficient fungal pathogen *Ssc* Δ*saxA* enriched only 5 taxa in Col‐0 WT (fdr‐adjusted *p* < 0.001, Figure 5a). All five—*Knoellia*, *Fluviicola*, *Pedobacter*, *Brevundimonas*, and *Devosia*—were also enriched in the ILZ during *Ssc* WT infection. Conversely, control PDB treatment did not lead to the enrichment of any bacterial taxa in the ILZ (a higher *p*‐value cutoff of *p* < 0.05 was used to detect any changes, Figure 5A), but rather had a suppressive effect, with significantly differently abundant bacterial groups showing a negative log2FC.

In the *A. thaliana* Col‐0 *myb28/29* mutant, which is completely deficient in the biosynthesis of aliphatic glucosinolates (aGLSs), the *Ssc* WT fungus enriched a total of 16 bacterial genera in the ILZ compared to the NILZ (*p* < 0.001, Figure 5B). Five of these 16 genera (*Pseudomonas*, *Simplicispira*, *Limnobacter*, *Rhizobacter*, and *Marmoricola*) were also differentially abundant in the Col‐0 WT/*Ssc* WT infection, but the enrichment overall was less strong in the *myb28/29* mutant (all but one of 16 genera with log2FC < 4.5). In the case of Col‐0 *myb28/29* plants infected with the *Ssc* Δ*SaxA* mutant, no bacterial genera were significantly differentially enriched between the ILZ and NILZ below *p* < 0.1 (Figure 5B). In the PDB treatment, 15 bacterial taxa showed a slightly higher abundance with a log2FC of at least 2 (at a higher *p*‐value cutoff of *p* < 0.05 was used to detect these changes, Figure 5B). Thus, *SaxA* influences the assembly of diverse leaf bacteria within the ILZ mostly, but not entirely, due to aITCs.

## 4 Discussion

Necrotophic fungal plant pathogens are highly destructive crop pests, causing massive crop losses under conducive conditions. Besides financial repercussions, the large‐scale application of pesticides to control them also has far‐reaching ecological consequences. Therefore, there is a strong incentive to develop alternative, more sustainable pathogen control strategies. *Sclerotinia sclerotiorum* (Ssc) has emerged as a model necrotrophic plant pathogen, in part due to its wide host range, including important crops. It has been suggested that bacterial biocontrol could be developed as an effective sustainable control strategy of Ssc (Han et al. 2023; Mülner et al. 2019). However, the traditional biocontrol discovery remains bottlenecked by inefficient, large‐scale screenings that often yield high false positives. Accelerating Ssc biocontrol development requires a deeper mechanistic understanding of *Sclerotinia sclerotiorum* (Ssc)‐bacterial interactions. While isothiocyanates (ITC) do exhibit antifungal properties, we focused on bacteria because of their known effects on Ssc (Savchuk and Dilantha Fernando 2004; Wang et al. 2024). Additionally, bacteria are easier to handle than many fungi, making them interesting for potential applications.

We find that Ssc WT infection strongly interacts with leaf bacterial diversity in the model plant *Arabidopsis thaliana* resulting in the enrichment of diverse bacterial taxa in the fungus infected leaf zone (ILZ). Effects of Ssc infection on the microbiome has not previously been thoroughly studied, although diseased lettuce stems were reported to have a relatively high amount of Gammaproteobacteria (Zhang et al. 2024). Changes to the host leaf microbiome caused by other eukaryotic pathogens has been well‐documented. Some biotrophic oomycete and fungal leaf pathogens, for example, elicit large‐scale changes on the host bacteriome of both infected (Agler et al. 2016; Jakuschkin et al. 2016) and systemic tissues (Goossens et al. 2023). Microbiomes of plants infected by necrotrophic pathogens are generally less well‐studied. In *Zymoseptoria tritici*, the wheat leaf blotch pathogen, infection reshaped microbiomes in both infected and systemic tissue, mainly in resistant wheat cultivars (Seybold et al. 2020). Leaf infection with *Botrytis cinerea*, which is closely related to Ssc and has a similar lifestyle, induced microbiome changes in systemic rhizosphere tissues in tomato and strawberry (De Tender et al. 2016; Rizaludin et al. 2025), but local tissues were not investigated. While we did not investigate the rhizosphere microbiome, we did not detect effects in systemic leaf tissues, which may suggest that the most profound effects of SSc infection are local.

Our results indicate that aliphatic glucosinolates (aGLS) play an important role in determining the structure of the bacteriome of Ssc‐induced necrotic lesions. This is consistent with previous work that suggests that remodelling and suppression of host immune metabolites by pathogens is an important mechanism shaping microbiomes. For example, infection of the obligate biotrophic oomycete *Albugo candida* alters the synthesis of indole glucosinolates in *Arabidopsis*, which allows increased colonization of non‐adapted pathogens (Prince et al. 2017). Effects of the wheat leaf blotch pathogen *Zymoseptoria tritici* on local and systemic bacteriomes of resistant wheat cultivars were also correlated to strong changes to benzoxazinoids (BXs), metabolites that function similarly to glucosinolates as phytoavengins (Kliebenstein and Kvitko 2023; Seybold et al. 2020). In particular, upon infection, a susceptible cultivar showed no changes to glycosylated BX pools and limited effects on the bacterial microbiome, while BXs in a resistant cultivar shifted to the free, toxic form, together with an altered microbiome (Seybold et al. 2020). Our results similarly suggest that the toxic breakdown product of aGLS, aliphatic isothiocyanates (aITCs), was important in microbiome remodelling. However, in this case, it appears that ITC *detoxification* by SSc allows bacteria to grow in necrotic tissue, while in the *Z. tritici* example the presence of the toxic breakdown product most likely altered the microbiomes. The difference could be due to differences in the effects of the breakdown products or due to other host traits, or both.

The broad enrichment of specific bacterial taxa within the ILZ mainly when ITCs are detoxified fits to previous work (Unger et al. 2024) showing that GLS breakdown products in Col‐0 are toxic to diverse commensal leaf bacteria. Many of the bacterial taxa identified in our dataset overlap with (e.g., *Pseudomonas*) or are closely related to taxa tested there. Because the SaxA hydrolase functions specifically to degrade host ITCs, this provides strong functional evidence *in planta* that these commensal genera are sensitive to lesion‐associated ITCs and are selectively rescued when the pathogen actively metabolizes and clears these plant chemical defences. This too is consistent with previous work showing in vitro that growth suppression of diverse commensal bacteria by aITCs can be relieved by ITC detoxification by *Pseudomonas viridiflava* SaxA (Mauri et al. 2026), suggesting the importance of ITC detoxification may cross kingdom borders. Thus, pathogen‐mediated ITC degradation could act as a crucial ecological driver, effectively opening a niche to opportunistic leaf commensals.

Interestingly, we observed that the fungal SaxA had a significant effect in both wild‐type plants and in *myb28/29* mutant plants, which do not produce aGLS and therefore cannot have aITCs. So far, SaxA has been characterized strictly as an isothiocyanate hydrolase and other substrates are not known. Thus, a likely explanation is that SaxA also plays a role in detoxifying indole ITCs, derived from indole GLS. Indole GLS are, in contrast to constitutively produced aGLS, largely induced upon infection. In addition, they are still produced in *myb28/29* plants because they are derived from tryptophan in a pathway independent of aGLS (Sønderby et al. 2007). Indeed, during Ssc infection the indole GLS pool decreases in infected tissue, consistent with their hydrolysis to ITCs (Chen et al. 2021). Ssc SaxA attacks both aliphatic and aromatic ITCs, but indolic ITCs have not to our knowledge been tested (Chen et al. 2020). Thus, we hypothesize that the effect of SaxA on bacteria in necrotic lesions is shaped by both aliphatic and indole GLS and ITCs.

Ssc infection in *A. thaliana* Col‐0 was also reported to cause accumulation of other anti‐fungal compounds and camalexin, among other plant metabolites. Interestingly, however, we found only weak effects on bacteria in necrotic lesions in the Col‐0 *myb2829* / Ssc Δ*SaxA* system compared to healthy tissue. The expected release of nutrients and the lack of plant immunity in necrotic tissue, as well as the lack of aliphatic glucosinolate‐related metabolites might be expected to greatly increase bacterial colonization in these tissues. One explanation could be that Ssc produces antibiotics or antimicrobial peptides. For example, both oomycete and fungal plant pathogens have been shown to produce antimicrobial effector proteins that target specific bacteria to promote virulence (Gómez‐Pérez et al. 2023; Snelders et al. 2021). Ssc secretes a range of effector proteins, but most of those characterized so far are only known to induce necrosis in hosts (Newman et al. 2023) and none have so far been described to affect plant microbiomes. If this were the case, however, we would expect to observe bacterial *suppression* in lesions in WT plants as well, where we mostly observed enrichment. While we cannot exclude antimicrobial production, our results are more consistent with the lack of microbiome effects in Ssc Δ*SaxA* lesions being due to accumulation of ITCs (aliphatic and indolic in WT plants and indolic in *myb28/29* plants). ITCs are toxic to a range of commensal leaf bacteria (Unger et al. 2024), and therefore may function as an efficient mechanism of clearing opportunistic bacteria from wounds, which could help prevent opportunistic infections.

It is worth noting that *myb28/29* mutants also exhibit altered iron homeostasis, which contributes to their sensitivity under various stress conditions independently of their aGLS deficiency. While the effects of both the fungal and host mutations together show that aGLS/aITCs play important roles in shaping the lesion bacteriome, it is possible that iron‐related or other physiological changes contribute to effects between the WT and *myb28/29* mutants (Coleto et al. 2020). Further experiments may be able to distinguish these by for example supplementing GLS or ITC directly back to the *myb28/29* mutants. Carrying such an experiment out in a realistic way, however, will require a better understanding of physiology of the mutant plants and the localized scales of GLS hydrolysis during pathogen attack.

In conclusion, the broad‐range necrotrophic fungal pathogen Ssc strongly rearranges the host microbiome in necrotic lesions. This rearrangement is almost completely dependent on detoxification of ITCs, the breakdown products of glucosinolates, demonstrating that interactions of host and pathogen factors together shape the bacteriome. It is unclear what role this rearrangement plays on the outcome of Ssc infection. However, bacteria recruited to leaves during pathogen infection can in some cases contribute to reduced fungal virulence (Goossens et al. 2023). Indeed, diverse bacteria including Actinomycetota (*Streptomyces*, *Micromonospora*), Bacillota (*Bacillus*) and Pseudomonadota (*Pseudomonas*, *Cupriavidus*) were shown to affect the growth of Ssc (Belt et al. 2025; Savchuk and Dilantha Fernando 2004; Liu et al. 2024; Estoppey et al. 2025). Thus, it is possible that Ssc bacteria may reduce Ssc virulence, for example via nutrient competition, or that they could increase virulence, for example by further inducing alterations to immune signals in neighbouring tissues. Thus, future work should focus on elucidating these effects.

## Author Contributions

**Shubhangi Sharma:** writing – original draft, methodology, conceptualization, investigation, data curation, visualization, validation. **Matthew Agler:** conceptualization, project administration, resources, supervision, writing – review and editing, funding acquisition, methodology. **Jingyuan Chen:** writing – review and editing, methodology, resources.

## Funding

This work was supported by Deutsche Forschungsgemeinschaft, EXC 2051 ‐ Projektnummer 390713860, 458884166 and Max‐Planck‐Gesellschaft.

## Conflicts of Interest

The authors declare no conflicts of interest.

## Supporting information


**Figure S1:** Detailed diagram of the experimental approach for investigating bacterial communities associated with *S. sclerotiorum*‐infected *A. thaliana* leaves.
**Figure S2:** Bacterial community analysis of *A. thaliana* Col‐0 *myb28/29* genotype infected with *S. sclerotiorum* Δ*SaxA*.

## Data Availability

All raw sequencing data is available under NCBI Project number PRJNA1508989 (https://www.ncbi.nlm.nih.gov/bioproject/PRJNA1508989). Processed data (ASV tables and metadata) and R code to generate the figures and Supporting Information are available at Figshare (10.6084/m9.figshare.30113110).
